# PSAIA – Protein Structure and Interaction Analyzer

**DOI:** 10.1186/1472-6807-8-21

**Published:** 2008-04-09

**Authors:** Josip Mihel, Mile Šikić, Sanja Tomić, Branko Jeren, Kristian Vlahoviček

**Affiliations:** 1 Department of Electronic Systems and Information Processing, Faculty of Electrical Engineering and Computing, University of Zagreb, Unska 3, 10000 Zagreb, Croatia; 2 Rudjer Bošković Institute, Bijenička 54, HR-10000 Zagreb, Croatia; 3 Bioinformatics Group, Department of Molecular Biology, Division of Biology, Faculty of Science, University of Zagreb, Horvatovac 102a, 10000 Zagreb, Croatia

## Abstract

**Background:**

PSAIA (Protein Structure and Interaction Analyzer) was developed to compute geometric parameters for large sets of protein structures in order to predict and investigate protein-protein interaction sites.

**Results:**

In addition to most relevant established algorithms, PSAIA offers a new method PIADA (Protein Interaction Atom Distance Algorithm) for the determination of residue interaction pairs. We found that PIADA produced more satisfactory results than comparable algorithms implemented in PSAIA.

Particular advantages of PSAIA include its capacity to combine different methods to detect the locations and types of interactions between residues and its ability, without any further automation steps, to handle large numbers of protein structures and complexes. Generally, the integration of a variety of methods enables PSAIA to offer easier automation of analysis and greater reliability of results.

PSAIA can be used either via a graphical user interface or from the command-line. Results are generated in either tabular or XML format.

**Conclusion:**

In a straightforward fashion and for large sets of protein structures, PSAIA enables the calculation of protein geometric parameters and the determination of location and type for protein-protein interaction sites. XML formatted output enables easy conversion of results to various formats suitable for statistic analysis.

Results from smaller data sets demonstrated the influence of geometry on protein interaction sites. Comprehensive analysis of properties of large data sets lead to new information useful in the prediction of protein-protein interaction sites.

## Background

Analysis performed on small data sets have shown [1] that geometrical properties of the protein surface influence protein-protein interactions. For the analysis of larger data sets (e.g. the non-redundant set of PDB structures), tools that can process a wider range of structural information descriptors are necessary.

It is also important to generate results that can be easily formatted for statistical analysis with standard software. Current software for protein analysis (standalone programs or web services) typically investigates a single aspect of molecular structure geometry and generates results only as simple text tables. These tables, whilst easily readable, are not ideal for parsing by computer.

Tools for determining protein-protein interactions are very scarce. Also, current software tools for structural property calculations from protein geometry (e.g. NACCESS [2]; DSSP [3]; DPX server [4]; CX server [5]) process only one molecular structure at a time. To avoid the need to manually run the program repeatedly for each structure, a user would need to write a controlling script in order to automate the analysis for a batch of structures.

PSAIA was designed both to simplify the use of algorithms for the analysis of sets of Protein Data Bank (PDB) [6] files implemented in other applications [7-9], and to provide access to algorithms for interaction analysis thus far not readily available to the public [10-12]. Additionally, we have included a novel algorithm for interaction analysis, PIADA (Protein Interaction Atom Distance Algorithm). PIADA defines interaction types according to the type of interacting atoms or groups of atoms as well as the residue distances (defined by the closest atoms). All interactions with the atom distance below the sum of their Van der Waals radii plus 1.125 Å are classified as Van der Waals, while interactions between the polar atoms at distance below 4.7 Å are classified as polar. Hydrophobic interaction is defined as an interaction between any of the so called hydrophobic amino acid residues (Alanine, Isoleucine, Leucine, Methionine, Phenylalanine, Proline and Valine) when their side chains are within the 4.7 Å distance. It is possible for one pair of the interacting amino acids to participate in multiple types of interactions. For details see the paragraph '**PIADA (Protein Interaction Atom Distance Algorithm**)' and Table 1.

**Table 1:** Definition of ionic and polar interactions utilized in PIADA. Protein interaction partners used in the PIADA algorithm for the definition of ionic and polar interactions. The hydrogen bonds are included in the latter, but due to the lack of information about directionality they are not explicitly specified.

Interaction type	Defined distance	Proton donors	Proton acceptors
Ionic	< 6Å	Lys N(NH3+) Arg N(NH2+) terminal N(NH3+)	Asp O(COO-) Glu O(COO-), terminal O (COO-)
Polar Group I	< 4.7Å	Asn N(H2) Cys S(H) Gln N(H2) His N(H) Ser O(H) Thr O(H) Trp N(H) Tyr O(H) N backbone	Asp O(COO-) Glu O(COO-)
Polar Group II	< 4.7Å	Lys N(NH3+) Arg N(NH2+)	Asn O (C=O) Cys S(H) Gln O(C=O) His N Ser O(H) Thr O(H) Tyr O(H) O backbone
Polar Group III	< 4.7Å	Asn N(H2) Cys S(H) Gln N(H2) His N(H) Ser O(H) Thr O(H) Trp N(H) Tyr O(H) N backbone	Asn O(C=O) Cys S(H) Gln O(C=O) His N Ser O(H) Thr O(H) Tyr O(H) O backbone

PSAIA can process each chain within a given molecular structure file separately (all other chains are ignored). This feature usefully allows easy investigation of structure features before and after association (in bound and unbound form), but is rarely offered in other software tools. Instead, users are required to create a separate PDB file for each chain they wish to investigate individually.

Four protein-protein interaction analysis algorithms are currently offered to the user by PSAIA. A strong feature of PSAIA is that it allows these algorithms to be used in combination with a selection of structure analysis methods. Whilst valuable, information provided by specialised databases, e.g. *i*PFAM [13] represents only one analytical approach. Using software tools such as PSAIA the user is able to vary the methods employed and also to analyse unpublished structure data.

PSAIA supports two output formats: a textual table, for reading directly, and XML (Extensible Mark-up Language) for parsing for further statistical analysis.

PSAIA can process nucleotide as well as peptide chains from PDB, although currently no interaction parameters are calculated for nucleotide chains.

## Implementation

PSAIA consists of two separate tools: the PSA (Protein Structure Analyser) and the PIA (Protein Interaction Analyser). The software is designed to function in both Linux and Windows operating systems, either as a command-line tool or through a standalone Graphical User Interface (GUI). The software is written in C++ and the GUI uses the QT Library 4.1.4 [14]. PSAIA uses standard Protein Data Bank (PDB) structure files in text format as input data, and provides output in both text (table) and XML formats. PSAIA is a modular tool that can be easily adapted to particular user requirements by simple editing of configuration files.

PSAIA verifies all input files format including configuration and PDB files. The input filter accepts "null" chains (chains without identification character) in PDB files. From summer 2007, PDB files with "null" chains will not appear in new releases. PSAIA accepts "null" chains for backward compatibility.

In case of missing parameters (e.g. atom types or residues used in PDB file, but not specified in PSAIA configuration files) PSAIA will issue a warning to the user at the end of processing. In case of incorrect PDB or configuration file formats, PSAIA will stop execution and display a warning message.

### Structure algorithms

Geometry calculation algorithms included in PSAIA work primarily at the atomic level, with residue attribute values derived by averaging atomic measurements. The following geometry parameters can be calculated: *Depth Index *– DPX [9], *Protrusion Index *– CX [8], *Solvent Accessible Surface Area *– ASA [7], *Relative Solvent Accessible Surface Area *– RASA [2]. PSAIA also associates a hydrophobicity value to each residue using the Kyte and Doolittle scale [15].

### ASA and RASA

The **accessible surface area **(ASA) is the atomic surface area of a molecule (protein, DNA, etc.) that is accessible to a solvent, and is usually expressed in Å^2 ^(square Ångstroms). ASA is calculated using the 'rolling ball' algorithm [16], which uses a sphere (representing the solvent) of a particular radius to 'probe' the surface of the molecule. A typical value of a 'probe radius' is 1.4 Å, which approximates the radius of a water molecule.

Relative ASA (RASA) attribute is the per-residue ratio between the calculated ASA and 'standard' ASA for a particular residue. Standard ASA values are usually determined by calculating ASA values of a central residue in a triplet (e.g. ALA-X-ALA) [2].

For use within PSAIA, for each of the twenty standard residues, one thousand random triplets (with the desired residue in the middle) were selected from random PDB files. For each central residue within the extracted triplet, five ASA attributes (given above) were calculated and their mean values were determined. These mean values are taken as representative standard ASA attributes for each of the twenty residues, and are provided in a file within PSAIA distribution. However, the user has the possibility to provide alternative 'standard ASA' values as a separate input file. Standard ASA values were also calculated for nucleotides in the same manner as for amino-acid residues.

The ASA and RASA parameters are calculated separately for each chain in the PDB file, as well as for all chains together. Compared to NACCESS there is a difference in calculation of backbone and side chain ASA of Glycine. PSAIA treats Glycine C-alpha atom as a part of the backbone, while NACCESS considers it a part of the side chain.

For ASA and RASA, the following residue attributes are calculated:

• *total *– sum of all atom values.

• *backbone *– sum of all backbone atom values.

• *side-chain *– sum of all side-chain atom values.

• *polar *– sum of all oxygen, nitrogen and phosphorus (for nucleic acids) atom values.

• *non-polar *– sum of all carbon atom values.

### DPX and CX

The depth of an atom *i (*DPX*i) *is defined as its distance (Å) from the closest solvent accessible atom *j *(atomic solvent accessibility, *asaj *> 0):

DPX*i *= min(*d*1, *d*2, *d*3, ..., *dn*)

where *d*1, *d*2, *d*3, ..., *dn *are the distances between the atom *i *and all solvent accessible atoms. The depth (DPX) is thus zero for solvent accessible atoms, and >0 for atoms buried in the protein interior, with deeply buried atoms having higher DPX values [9].

The principle of CX algorithm is as follows. For each non-hydrogen atom in protein structure, the program calculates the number of heavy atoms within a fixed distance R (the default value is 10 Å). The number of atoms within sphere is multiplied by the mean atomic volume found in proteins (20.1 ± 0.9 Å; [17]), which gives the volume occupied by a protein within sphere V_int_. The remaining volume of the sphere, V_ext_, is calculated as the difference between the volume of the sphere and V_int_. The CX value is then defined by V_ext_/V_int _[8].

For both DPX and CX the following residue attributes are calculated:

• *total mean *– mean value of all atom values.

• *total standard deviation.*

• *side-chain mean *– mean value of all side-chain atom values.

• *side-chain standard deviation.*

• *maximum *– highest of all atom values.

• *minimum *– lowest of all atom values.

### Interaction algorithms

The following interaction algorithms are implemented in PSAIA (subprogram PIA):

• *Atom Nucleus Distance *– method introduced by Ofran and Rost [11].

• *Atom Van der Waals Radii Distance *– method introduced by Aytuna et al. [18].

• *ASA Change on Complexation *– method introduced by Jones and Thornton [1].

• PIADA – a novel algorithm designed during PSAIA development.

PIA calculates only protein-protein interaction. Protein-nucleic acid and nucleic-nucleic acid interactions are not included in the calculation.

### Atom Nucleus Distance

This very simple method defines two residues from the opposite chains as interacting if there is at least one pair of non-hydrogen atoms, one from each residue, at a distance below the specified threshold. Values for the threshold are usually 4.5 – 6 Å.

### ASA Change on Complexation

In this method, ASA is calculated for a particular residue before and after the process of complexation. If the difference between ASA in unbound and bound form is above the specified threshold, then a residue is defined as an interacting residue. The usual value for threshold is 1 Å^2^. It is important to emphasize that this method can only define interaction residues, but not their interacting partners.

### Atom Van der Waals Radii Distance

Two residues from the opposite chains are marked as interacting if there is at least one pair of non-hydrogen atoms, one from each residue, at a distance smaller than the sum of their van der Waals radii plus a threshold. Thresholds are usually 0.5 – 1.5 Å.

### PIADA (Protein Interaction Atom Distance Algorithm)

Although algorithms that use atomic distance cut-off for definition of interaction exist, they are either too general [18,19] or do not contain [13] hydrophobic and long range electrostatic interactions. While designing PIADA, we directed our attention to possible atom and residue interaction partners (only for hydrophobic).

Our algorithm for detecting the protein-protein interactions calculates four different types of interactions: ionic, Van der Waals, hydrophobic and polar. Table 1 shows decision criteria for ionic and polar interactions used in PSAIA. Many of these interactions can be classified as hydrogen bonds, however currently we do not consider directionality and therefore have not defined this type of interaction explicitly. Nevertheless, pre-processing of the raw PDB file by adding hydrogen atoms and selecting the best amino-acid residue rotamers would enable reliable analysis of the hydrogen bond interactions.

*Hydrophobic interactions *are defined as interactions between any two of the seven non-polar amino acids (Alanine, Isoleucine, Leucine, Methionine, Phenylalanine, Proline and Valine) when the following condition is fulfilled: distance between residues, measured as distance between any two atoms from different residues, is less than 4.7 Å.

*Van der Waals interactions *are defined as interactions between any two amino acids when the distance between residues (non-hydrogen atoms) is less than the sum of their Van der Waals radii plus 1.125 Å. The value of 1.125 Å has been determined empirically by comparing our results with results from the *i*PFAM database [13].

### Graphical user interface

**Figure 1:**
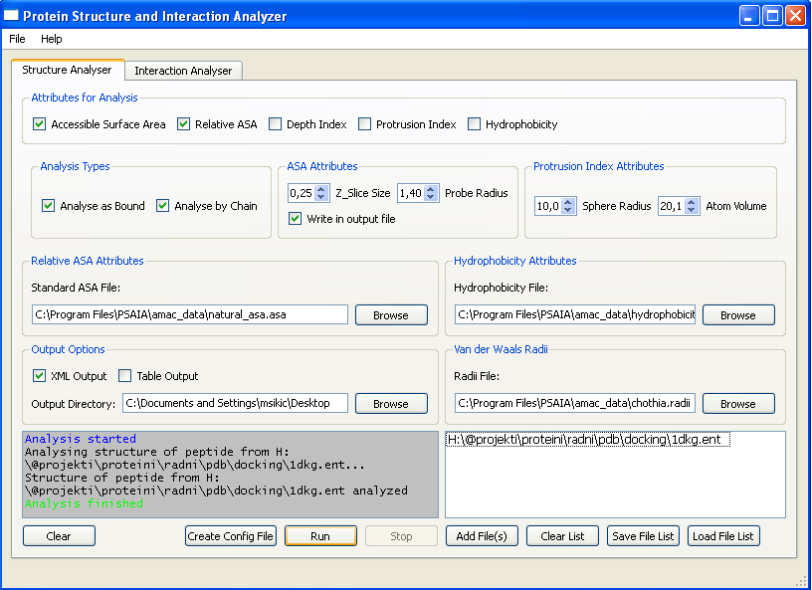
**PSAIA – Structure analyser**. Figure shows GUI structure analyser part of PSAIA.

**Figure 2:**
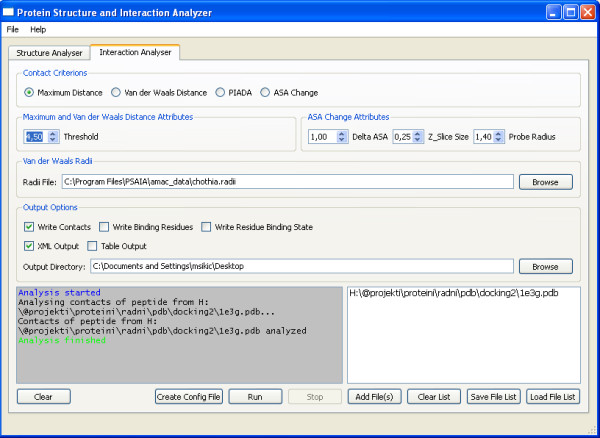
**PSAIA – Interaction analyser**. Figure shows GUI of interaction analyser part of PSAIA.

There are two main tabs in the interface: the structure analyser and the interaction analyser. Figure 1 presents the structure analyser and Figure 2 the interaction analyser.

### Structure analyser

Prior to analysing a structure, the user should predefine the following files:

• A file with total, backbone, side chain, polar and non-polar ASA for particular amino acid residue and nucleotides.

• A file with value of hydrophobicity for each of amino acid residues.

• A file with Van der Waals Radii for each atom of particular residue and nucleotide, ligand atoms and hetero atoms.

All of the files mentioned above are included in the installation package with common values, but users can easily change them or add some values if it is necessary for their analysis. The software memorises the location of the last configuration files and uses them as default for the next run.

The user can optionally change ASA attributes (z slice size and solvent probe radius) [7] and protrusion index attributes (sphere radius and atom volume) [8]. Solvent probe radius, sphere radius and atom volume are described in paragraph '**Structure algorithms**', while with z-slice value users can define precision of calculation. It is recommended to use default values.

Upon predefining parameters, the user should select the following: output directory attributes for analyses (ASA, RASA, DPX, CX and hydrophobicity), chain bonding type (bound and/or unbound) and output data format (text table and/or XML). Example output from structure analyzer is given in Additional files 1 and 2.

The final step is the selection of protein files. Files can be added manually or from a predefined list file. PDB files should be correctly formatted or the software will return an error message.

There are two additional options: saving the already selected files in a list file and creating a configuration file based on selected parameters and definition files. Both of these options can also be used for a console version of application for massive analysis on the remote server.

### Interaction analyser

Interaction analyser gives the user a possibility to use one of the four interaction calculation algorithms mentioned above (maximum distance, Van der Waals Distance, ASA change and newly developed PIADA algorithm). Like in the structure analyser, the user should first specify parameters and configuration file. The only configuration file is Van der Waals Radii file. In accordance with the selected algorithm, the user can choose threshold distance (maximum distance or Van der Waals distance algorithm) or delta ASA threshold value [1] between unbound and bound residue, z slice and probe radius value (delta ASA algorithm). Secondly, the user chooses the output directory, output format (table and/or XML) and output data options (contacts – interaction residue pairs, only binding residues and binding status for each residue; all output combinations are given as example in Additional files 3, 4, 5, 6, 7, 8, 9, 10, 11, 12, 13). Finally, the user selects protein files, starts application and has the possibility to create a file list and configuration file for console application.

### Console applications

There are two console applications: PSA (protein structure analyser) and PIA (protein interaction analyser). In order to use both of them, it is necessary to define the configuration file and a file with the list of proteins. These files can easily be created by GUI version of the application.

## Results and Discussion

PSAIA is a software tool that integrates several algorithms for protein interaction and structure geometry analysis of protein complexes into a single application. The software can be used via a graphical user interface or from the command-line, and user can choose between a tabular and XML output formats. Protein structure and protein interaction data are in many cases investigated together, and respective algorithms often share the same software code. Therefore, it was an obvious choice to combine the two analysis types into a single application.

The main aim of PSAIA is to analyse and predict both protein structural features and interaction sites. XML formatted output simplifies conversion of results into any other desired format. GUI and command-line versions for MS Windows and Linux platform make the software accessible to a broader range of potential users. Furthermore, the console application enables the execution of large scale analyses on remote servers.

Results for each of the two integrated tools (PSA and PIA) are presented separately.

### Structure analyser

A single program that contains the same set of algorithms is not available, so we compared our results with those obtained by original software tools for each separate algorithm. NACCESS and DSSP are the most frequently used algorithms for ASA calculation. Both are available only with the command-line interface. For testing purposes, we used a RAS-RAL crystal structure (PDB:1LFD). The obtained values (Additional files 1, 2, 14, and 15) are similar. Overall protein ASA calculated by NACCESS is 22 523.6 Å^2^, by DSSP 22 704 Å^2 ^and by PSAIA 22 399 Å^2^. The differences are very small, up to 1 percent. When we test the performance for 336 proteins from protein-protein docking benchmark [20], the overall ASA calculated by NACCESS is 4404902.1 Å^2^, by DSSP 4455022 Å^2 ^and by PSAIA 4357359.26 Å^2^. The differences between methods are similar to those calculated for PDB:1LFD – up to +/-2 percent. Most of the discrepancies originate from different rounding off procedures, PSAIA rounds off at the third decimal place, NACCESS at the second and DSSP at the first. DSSP only calculates total ASA per residue, while NACCESS and PSAIA also calculate backbone, side chain, polar and non-polar ASA. The main advantage of PSAIA is that it can perform calculations on chains in unbound and bound conformations, which is not possible with other available tools.

DPX and CX values were calculated using web servers [4,5]. Results for PDB:1LFD, calculated by PSAIA (Additional files 1, 6, and 16) differ from those obtained with web servers (Additional files 17 and 18) by up to +/-2 percent. Web servers are intended for calculations of a single PDB file at a time and have no possibility of automating the calculation for a batch of structures. Instead, the user should repeatedly set calculation parameters and upload new files. The main advantage of PSAIA is the possibility to sequentially process a large number of files in one step. Also, CX server produces results only for unbound chains while PSAIA gives the possibility to view results for both unbound and bound forms. DPX and CX servers output tabulated results for each atom in the PDB file. However, we found this option unnecessary for large scale analyses and instead designed PSAIA to output per-residue maximum, minimum, mean and standard deviation values. In addition, side chain, backbone, polar and non-polar parts of residue are given as separate values – a feature we consider informative for the analysis.

From the above results, it is evident that the precision of PSAIA calculation is comparable to that of the established tools, while the easy handling provided by the GUI, the possibility of calculation of bound and unbound chains and "batch" processing a large number of input files are PSAIA's advantages.

We tested PSAIA performance with all algorithms and output data file format included. The test platform was a server with Intel Xeon Dual Core 5140, 2.33 GHz processor. The test set consisted of 336 proteins.

The average running time for PSA was 708 seconds (2s per protein), and PIADA algorithm of PIA ran for an average of 42 seconds (0.1 s per protein).

### Interaction analyser

The interaction analyser consists of three pre-existing algorithms for calculation of interacting residues plus the newly designed PIADA which determines the interaction between particular residues according to their type and the type of interacting atoms as described in the implementation section. A similar algorithm is used for creating iPFAM database [13]. However, iPFAM can only provide information, generated in one way, describing the PDB structures from which it was constructed. It cannot include information about interaction residue pairs outside known domains. PIADA can be applied in an intuitive fashion to new protein complexes obtained experimentally or by computer simulation, e.g. docking. With PSAIA, the user can compare results obtained by different algorithms. This enables a more precise calculation of a particular interaction than with the distance based algorithms [11,18] and the algorithm based on ASA change [1] alone.

To test the new algorithm, we compared the number of interactions obtained by PIADA on a set of 2 445 protein complexes with the results obtained by the distance based algorithm (with threshold distance of 4.5 Å) and data from the iPFAM database (version 19, August 2006).

Total numbers of interacting residues found by a particular algorithm are summarised in Table 2. According to the number of detected interacting residues, the highest score was obtained by the algorithm based on delta ASA (439 115), followed by PIADA (394 356) and the atom distance algorithm (374 805). The least of the residues were found in iPFAM database (334 640). One of the reasons for this is the fact that iPFAM database consists only of interaction pairs from known PFAM [21] protein domains.

**Table 2:** Comparison of PIADA with other algorithms. This table shows comparison between the number of protein interacting residues found by PIADA and either by 'maximal distance method', 'delta ASA method' or interactions present in iPFAM. One of the reasons for the large discrepancy in the results obtained by PIADA and by iPFAM database is the fact that iPFAM results cover only the residues that are inside PFAM domains.

PIADA	Number of residues found by both algorithms	Number of residues not found by other algorithms	Number of residues not found by PIADA
Atom distance	382 175	12 181	5 051
*I*PFAM (Inside PFAM domains)	259 649	134 707	74 991
Delta ASA	387 187	7 169	51 928

Interacting residues detected by the atom distance algorithm overlap with PIADA results in 97 percent of instances. The observed difference occurs mostly in the 'weak Van der Waals' interactions, i.e. those with inter-atomic distance under 4.5 A, but above the Van der Waals threshold (see Table 1 and the definition of hydrophobic and Van der Waals interactions above). PIADA found 78 % of the total number of interacting residues present in iPFAM database. However, PIADA detected many interacting residues not present in iPFAM. Possible explanations are a) longer distance threshold used for ionic and polar interactions, and b) taking into account the hydrophobic interactions not covered by iPFAM database. Further reasons for the difference in the results obtained by these two algorithms are in different threshold value used for definition of the Van der Waals interactions (1.125 Å in PIADA; 1.5 Å in *i*PFAM). PIADA found 88 % of the interaction residues found by the ASA based algorithm. The main difference between these two methods lies in different definitions of interaction (distance based and space based). Also, delta ASA (difference between ASA in unbound and bound form) threshold for interaction is taken as 1 Å^2 ^[10]. More careful tuning of this parameter would probably lead to better correspondence of results, however that is outside the scope of this paper.

Comparing the results from the above mentioned algorithms, we found that PIADA and 'delta ASA' algorithms detected the largest number of interaction residues confirmed by at least one other method.

To our knowledge, there is no other software tool that calculates protein interaction residues and pairs for a large number of proteins in automated fashion, and users are often forced to use some form of scripting for large-scale batch analysis.

### Tool Applicability

We developed PSAIA in order to predict and investigate protein interaction sites. We used the program on a series of 336 non-redundant 3D structures of protein complexes in order to obtain structural information relevant for predicting protein interaction sites. The results obtained in combination with sequence information enabled us to develop a highly reliable method for predicting protein interaction sites (manuscript in preparation). We successfully tested the method on a test set of 1387 PDB structures prepared by PSAIA.

Moreover, PSAIA is of great help as a verification tool in docking studies. For example, we use it for the calculation of changes in ASA as well as for a quick scan of interacting residue pairs in docked structures.

### Further work

In the next version we plan the following improvements to the software: increasing the number of available algorithms and improving implementation of the existing ones in terms of speed; program parallelisation and support for other input data formats. Protein secondary structure is an important factor for studying protein interactions and therefore we plan to include one of the secondary structure calculation algorithms into the program PSAIA is designed for analysis of a large number of molecular structures using PDB files as input data. Further improvement in speed and processing capability of the program will be parallelisation of the analysis process by distributing one structure per processor. The next release of the PIADA algorithm will include the possibility to investigate interactions between molecules of any type, including hetero atoms and nucleic acids. Additionally, mmCIF and XML file formats are now widely used for storing molecular structure information in the Protein Data Bank, and the next program version will include the possibility to process these file formats.

## Conclusion

PSAIA can process a series of PDB files and calculate a large number of protein structure parameters as well as determine interaction residues based on several different algorithms. In addition PSAIA can calculate parameters for protein chains in unbound and bound form.

In addition to established algorithms, PSAIA offers our new PIADA algorithm for calculation protein interaction residue pairs. Results obtained from PIADA correlate well with, and generally improve upon, the results of the alternative algorithms. PIADA offers the advantage that it can be also used to define the type of protein interaction and to execute a quick scan of interacting residue pairs in docked structures.

Uniquely, PSAIA allows simultaneous application of so many different algorithms for structure analysis and for determination of interacting residue pairs. XML output enables an easy conversion of data to formats suitable for statistic analysis using standard software. Results from smaller data sets demonstrated the influence of geometry on protein interaction sites. Comprehensive analysis of properties of large data sets lead to new information useful in the prediction of protein-protein interaction sites.

## Availability and requirements

• **Project name: **PSAIA

• **Home page: **

• **Operating systems: **MS Windows, Linux

• **Programming language: **ANSI C++

• **Other requirements: **Qt 4.1.4 for Linux

• **License: **GNU GPL

• **Availability: **Additional file 19 contains MS Windows installer and Additional file 20 includes installer for Linux operating systems. Source code and updated installation versions are available from the project home page

• **Restrictions for the use by non-academics: **None

## Abbreviations

**PSAIA**: Protein Structure and Interaction Analyzer; **PSA**: Protein Structure Analyzer – console application; **PIA**: Protein Interaction Analyzer – console application; **PDB**: Protein data bank; **ASA**: Accessible surface area; **CX**: Protrusion index; **DPX**: Depth index; **MPI**: Message Passing Interface; **PVM**: Parallel Virtual Machine; **GUI**: Graphical User Interface; **XML**: Extensible Mark-up Language

## Authors' contributions

JM contributed to design, developed software and has been involved in manuscript drafting. MS contributed to design, had made intensive testing of software and was involved in manuscript drafting. ST contributed to design of PIADA algorithm, and was involved in revising the manuscript. BJ contributed to design and was involved in revising the manuscript. KV contributed to design and was involved in revising the manuscript. All authors read and approved the final manuscript.

## Supplementary Material

Additional file 1 PDB:1LFD – output of PSAIA Structure analyser in table form. This file includes information about ASA, DPX, CX and hydrophobicity values per residue of PDB:1LFD obtained by PSAIA. In this case, the chains were calculated in bound form. Click here for file

Additional file 2 PDB:1LFD – output of PSAIA Structure analyser in XML form. This file includes information about ASA, DPX, CX and hydrophobicity values per residue of PDB:1LFD obtained by PSAIA. In this case, the chains were calculated in bound form. Click here for file

Additional file 3 PDB:1LFD binding residues – output of PSAIA Interaction Analyser in table form. This file includes information about residues that are included in interaction in PDB:1LFD obtained by maximum distance algorithm from PSAIA application. Click here for file

Additional file 4 PDB:1LFD binding residues – output of PSAIA Interaction Analyser in XML form. This file includes information about residues that are included in interaction in PDB:1LFD obtained by maximum distance algorithm from PSAIA application. Click here for file

Additional file 5 XML Schema for binding residues. This is the XSD schema definition for XML output file (Additional file 6) Click here for file

Additional file 6 PDB:1LFD binding status – output of PSAIA Interaction Analyser in table form. This file includes information on interaction status (in interaction or not in interaction) of a particular residue in PDB:1LFD obtained by maximum distance algorithm from PSAIA application. Click here for file

Additional file 7 PDB:1LFD binding status – output of PSAIA Interaction Analyser in XML form. This file includes information on interaction status (in interaction or not in interaction) of a particular residue in PDB:1LFD obtained by maximum distance algorithm from PSAIA application. Click here for file

Additional file 8 XML Schema for binding status. This is the XSD schema definition for XML output file (Additional file 9) Click here for file

Additional file 9 PDB:1LFD contacts – output of PSAIA Interaction Analyser in table form. This file includes information on interaction partners in PDB:1LFD obtained by maximum distance algorithm from PSAIA application. Click here for file

Additional file 10 PDB:1LFD contacts – output of PSAIA Interaction Analyser in XML form. This file includes information on interaction partners in PDB:1LFD obtained by maximum distance algorithm from PSAIA application. Click here for file

Additional file 11 XML Schema for residue contacts. This is the XSD schema definition for XML output file. Click here for file

Additional file 12 XML Schema for peptide structure in bound form. This is the XSD schema definition for XML output file. Click here for file

Additional file 13 XML Schema for peptide structure in unbound form. This is the XSD schema definition for XML output file. Click here for file

Additional file 14 PDB:1LFD – output of DSSP application. This file includes information about secondary structure and total ASA per residue of PDB:1LFD obtained from DSSP application. Click here for file

Additional file 15 PDB:1LFD – output of NACCESS application. This file includes information about total, relative, backbone, side-chain, polar and non-polar ASA per residue of PDB:1LFD obtained from NACCESS application. Click here for file

Additional file 16 PDB:1LFD – CX output of PSAIA Structure Analyser. This file includes information about CX per residue of PDB:1LFD obtained by PSAIA. In this case, the chains were calculated in unbound form. Click here for file

Additional file 17 PDB:1LFD – output of CX server . This file includes information about maximum, minimum and average CX values per residue of PDB:1LFD obtained from CX server. Click here for file

Additional file 18 PDB:1LFD – output of dpx server . This file includes information about maximum, minimum and average CX values per residue of PDB:1LFD obtained from CX server. Click here for file

Additional file 19 PSAIA – MS Windows setup file. Setup file for installation PSAIA, PSA and PIA. Newer versions of the application can be downloaded from the project web site. Click here for file

Additional file 20 PSAIA – Linux installer. PSAIA installer for a x86 Linux platform. Linux installers for other architectures as well as the source code can be downloaded from the project web site. Click here for file
